# The Latent Dimensionality of Physical and Technical Performance Across Three Youth Soccer Tiers

**DOI:** 10.3390/jfmk11020177

**Published:** 2026-04-28

**Authors:** Adem Preljević, Saša Bubanj, Dušan Stanković, Miladin Okičić, Dalila Preljević, Emilija Petković, Miodrag Kocić, Tomislav Gašić, Bojan Bjelica, Ivana Parčina, Sanja Krsmanović Veličković, Milan Mihajlović, Tatiana Dobrescu, Adrian Mihai Sava

**Affiliations:** 1 Department of Biochemical Science and Sport, State University of Novi Pazar, 36300 Novi Pazar, Serbia; apreljevic@np.ac.rs; 2 Faculty of Sport and Physical Education, University of Niš, 18000 Niš, Serbia; dukislavujac@gmail.com (D.S.); miladinokicic.2001@gmail.com (M.O.); petkovicemilija@yahoo.com (E.P.); miodrag.kocic73@gmail.com (M.K.); gasictomislav@yahoo.com (T.G.); 3 Department of Philosophical Sciences, State University of Novi Pazar, 36300 Novi Pazar, Serbia; dpreljevic@np.ac.rs; 4 Faculty of Physical Education and Sport, University of East Sarajevo, 71420 Pale, Bosnia and Herzegovina; vipbjelica@gmail.com; 5 Faculty of Sport, University “Union-Nikola Tesla”, 11000 Belgrade, Serbia; ivana.parcina@fzs.edu.rs (I.P.); sanja.krsmanovic@fzs.edu.rs (S.K.V.); milan.mihajlovic@fzs.edu.rs (M.M.); 6 Department of Physical Education and Sport Performance, Vasile Alecsandri University, 600115 Bacau, Romania; sava.adrian@ub.ro

**Keywords:** youth athletes, soccer, anthropometry, motor control, development

## Abstract

**Objectives**: This study aimed to examine the structure of anthropometric characteristics, motor skills and specific motor skills in young football players. **Methods**: Study participants (427 male football players) were divided into pre-pioneers (11–13 y), *n* = 133; pioneers (13–15 y), *n* = 160; and cadets (15–17 y), *n* = 134. The entire sample of subjects was evaluated using 13 anthropometric and seven motor variables. The factor structure for each chronological age group was determined using Hotelling’s method. **Results**: Anthropometric characteristics showed three extracted factors in the pre-pioneers group, four factors in the pioneer group and two factors in the cadet group. Motor skills displayed three factors for the youngest group, two factors for the pioneers and three factors for the cadet group. Four factors were determined for specific motor skills in pre-pioneers, four in pioneers and three in cadet age. **Conclusions**: This study revealed structural variability and non-uniformity in the latent dimensions across age groups, with the total number of factors fluctuating between two and four. This study revealed two consistent latent dimensions in anthropometric data across all age groups: general morphological parameters and subcutaneous fat tissue. In motor skills, an initial universal factor is separated into central and energetic regulation of movements. Finally, specific motor skills demonstrated a transition from a highly differentiated four-factor structure in younger players toward a more integrated functional system in the oldest cohort, comprising intermuscular coordination, running speed with and without a ball; segmental speed of the lower extremities with a ball; and explosive force in hitting a ball with the foot and head.

## 1. Introduction

Basic motor skills serve as the foundation for teaching, extracurricular activities, and physical training, impacting the anthropometric characteristics of pupils, students, and athletes when applied systematically and consistently [1]. Specific motor skills are actions aimed at achieving a particular goal, requiring the coordinated use of multiple motor abilities within a specific context and time frame [2]. As these skills can be either innate or acquired [3], their development can be influenced to varying extents. In football, fundamental and specific motor skills allow players to perform complex movements with accuracy and effectiveness, impacting their capacity to execute both offensive and defensive roles [4]. For young players, the structured development of these skills is essential, as they provide the groundwork for more advanced techniques [5]. Training programs that enhance explosive power and specific motor skills can greatly improve players’ technical performance, physical fitness, and injury prevention [6].

While anthropometric traits establish the physical foundation, explosive power remains the primary determinant of on-field technical efficiency. Power, explosive strength, and balance play a more critical role in football success than anthropometric characteristics [7]. These abilities support nearly every movement a player makes on the field, from sprinting, jumping, tackling, and maintaining stability during rapid changes in direction. Upper body strength is also correlated with the explosive power of the lower body in football players [8], as arm swing and upper-body stability biomechanically amplify ground reaction forces during jumping and sprinting. Moreover, strength is essential for players to win physical duels, hold off opponents, and maintain power in shots and passes. Balance is correlated with shooting ability in football players under 18 years [9]. Additionally, balance allows players to remain stable under pressure, as well as to reduce injury risks [10,11].

Valuable insights into the interplay between physical development, motor skill and specific motor skill acquisition, ultimately contributing to more effective training strategies and improved performance outcomes for young football players, are always welcomed by the experts in the field. Petrić [12] administered situational motor tests to junior players from Vojvodina’s first and second federal leagues, assessing explosive power, precision, and agility. Using game performance as the criterion variable (physical and technical attributes, tactical execution, decision-making, team play contributions, and game-specific psychological attributes), regression analysis revealed that tests closely resembling football game elements were the strongest predictors of success.

The recent literature has increasingly highlighted the importance of continuous monitoring of anthropometric and motor variables across different age groups and regional contexts to better understand player development [13,14]. Therefore, it is necessary to further examine these relationships through more robust multivariate frameworks. This study aims to explore the latent dimensionality of anthropometric and motor variables in young football players of different age categories. By investigating the underlying factors that determine physical and technical performance across these age groups, this study seeks to provide valuable insights for coaches and practitioners to optimize training programs and talent identification strategies. Furthermore, a cross-sectional approach is utilized to identify a comprehensive cross-sectional profile of these variables, allowing for the detection of stable latent dimensions.

This research aimed to investigate the structure of anthropometric characteristics, motor skills and specific motor skills in young football players, examining how these factors vary with chronological age. By analyzing data from different age groups, we seek to identify key patterns and relationships that can guide the development of age-appropriate training interventions. Identifying latent dimensions provides a more rigorous framework for understanding the underlying constructs that underlie athletic performance. Factor analysis thus provides significant methodological value by multiple manifest variables into fundamental domains, allowing for a more precise characterization of the structural organization of talent across different youth categories.

We hypothesized that the structure of anthropometric characteristics, motor skills, and specific motor skills in young football players would differ across age groups, with identifiable latent dimensions that align with developmental and training stages.

## 2. Materials and Methods

### 2.1. Participants

This cross-sectional research was conducted with a population of young football players from the region of Western Serbia, specifically from five clubs: F.K. Borac Čačak, F.K. Radnički Kragujevac, F.K. Sloga Kraljevo, O.F.K. AS Novi Pazar, and F.K. Bane Raška. The sample was defined as a population of young football players of different chronological ages who were previously selected by their respective football clubs. The inclusion criteria were outlined as follows: male adolescents aged 11–17 years, level of training experience of at least 2 years, and undergoing a minimum of 3 weekly training sessions. Participants were examined for any injuries that could limit their ability to participate in the study. Exclusion criteria included individuals with cardiovascular, respiratory, or other medical conditions, and individuals with recent musculoskeletal injuries within the last three months. These players are actively involved in the training and competitive processes of the top five teams from the previous league cycle in the Quality League of Western Serbia, Morava Group.

**Figure 1:**
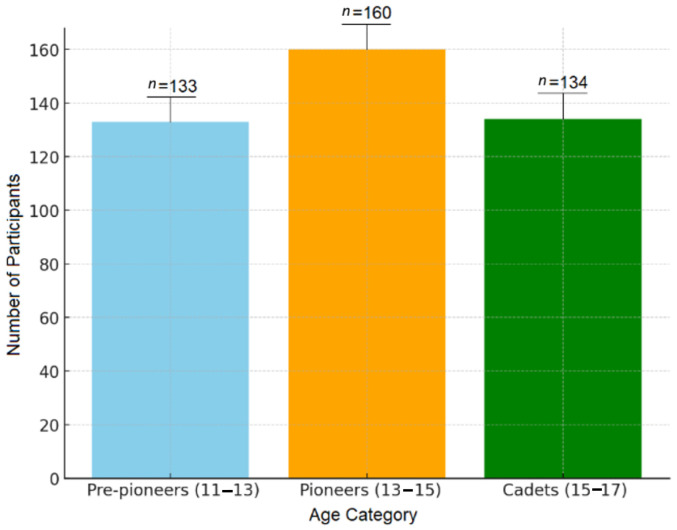
Participants included by age category in youth football clubs.

A cluster sampling approach was utilized, including a total of 427 members of youth football teams, divided into three sub-samples—pre-pioneers (body height = 158.9 ± 5.9 cm; body weight = 46.6 ± 5.9 kg), pioneers (body height = 169.5 ± 5.2 cm; body weight = 55.5 ± 6.6 kg), and cadets (body height = 174.6 ± 4.9 cm; body weight = 62.3 ± 8.1 kg)—according to age category: under-13 (up to 13 years old), under-15 (14 and 15 years old), and under-17 (16 and 17 years old), respectively (Figure 1). The classification of players was made according to the Rulebook of the Serbian Football Association that was in effect at the time of the research. All participants were male.

Participants signed informed consent forms for involvement in the study. By signing the form, the club, the parents and the participants confirmed that they were familiar with the experimental program. The study was approved by the Institutional Review Board of the Faculty of Physical Culture, University of “St. Cyril and Methodius”, Skopje, number 0201-488/4.

### 2.2. Sample of Variables

The entire sample of subjects was evaluated using 13 anthropometric variables, 7 motor variables, and 23 specific motor variables. These variables have been shown in previous studies to representatively assess each of the segments covered in this research [15]. Measurements of anthropometric characteristics were conducted first, followed by measurements of motor skills, while measurements of specific motor skills were conducted at the end.

#### 2.2.1. Instruments for Assessing Anthropometric Characteristics

For assessing the longitudinal dimension of the skeleton: body height (in cm); left arm length (in cm); left leg length (in cm). For assessing the circular dimension and body mass: thigh circumference (in cm); calf circumference in cm; body mass in kg. For assessing the transversal dimension of the skeleton: hip joint diameter (in cm); knee joint diameter (in cm); ankle joint diameter (in cm). For assessing subcutaneous fat: skinfold thickness of calf (in cm); skinfold thickness of abdomen (in cm); skinfold thickness of thigh (in cm); skinfold thickness of back (in cm). These variables represent all primary morphological domains, ensuring sufficient variance for a factor solution.

#### 2.2.2. Instruments for Assessing Motor Skills

For assessing the factor of energetic regulation of movements: Trunk Lift for 30 s (the number of correct trunk lifts completed in 30 s is recorded); Push-Ups on the Floor for 30 s (the number of correct Push-Ups completed in 30 s is recorded); Flexed-Arm Hang (in s); Standing Long Jump (in cm). For assessing the factor of central regulation of movements: Trunk Flexion (in cm); Single-Leg Tapping for 20 s (the number of correctly performed cycles in 20 s is recorded); Single-Leg Balance with Closed Eyes (the total time in seconds) from all three attempts is recorded (maximum of 180 s) [16]. Integrating basic motor skills justifies the physical dimension required for the execution of specific motor skills.

#### 2.2.3. Instruments for Assessing Specific Motor Skills

For assessing the specific motor skills: Horizontal Accuracy 15 m (a hit in the central circle is worth 5 points, in the second circle 3 points, and in the third circle 1 point), Horizontal Accuracy 20 m (expressed as previous), Horizontal Accuracy 25 m (expressed as previous), Vertical Accuracy—Hoop (the ball that passes through the hoop scores 5 points; the ball that hits the hoop scores 3 points; and the ball that hits the square, i.e., the rope lowered from the goalpost, where the hoop is located scores 1 point), Vertical Accuracy—Goal (hitting the small goal earns 5 points, the post of the small goal earns 2 points, and the post of the big goal earns 1 point), Single-Leg Ball Juggle (two correct attempts are measured, and the better result is used for analysis; the test duration is 20 s), Alternating Leg Ball Juggle (expressed as previous), Sprint with Ball 20 m (in s), Sprint with Ball 20 m Flying Start (in s), Sprint 20 m (in s), Slalom with Ball (in s), Slalom without Ball (in s), Semi-Circle Run with Ball (in s), Semi-Circle Run without Ball (in s), Dominant Leg Wall Pass (the number of correctly bounced balls in 20 s is counted), Non-Dominant Leg Wall Pass (expressed as previous), Alternating Leg Wall Pass (expressed as previous), Dominant Leg Parabolic Wall Pass (expressed as previous), Non-Dominant Leg Parabolic Wall Pass (expressed as previous), Alternating Leg Parabolic Wall Pass (expressed as previous), Dominant Leg Explosive Power (in m), Non-Dominant Leg Explosive Power (in m), and Head Explosive Power (in m) [17].

### 2.3. Procedures

During the project execution, measurements of anthropometric characteristics were conducted first, followed by measurements of motor skills.

All measurements were conducted within a single day for each participant group to maintain consistent environmental conditions. Prior to data collection, a familiarization session was conducted to ensure that all participants fully understood the testing protocols and instructions for the motor and specific motor skills. All measurements were taken under relatively similar temporal, spatial, and climatic conditions to ensure consistency and objectivity. Each participant had their relevant anthropometric points and levels accurately determined before measurements. Proper control was implemented during the reading and recording of results to prevent errors. The instruments used were of standard manufacturing, calibrated before each measurement session to ensure accuracy. Following the anthropometric measurements, motor and specific motor skills were assessed. To ensure the reliability, each motor test followed a standardized protocol: participants performed one practice trial and two (or three) official attempts, with the best result recorded for analysis. Rest intervals between attempts were maintained at 1 min and 5 to 10 min between different testing stations. Prior to the testing sessions, all participants underwent a standardized 15-min warm-up protocol. This consisted of 5 min of low-intensity running, followed by 5 min of dynamic stretching (focusing on major muscle groups), and 5 min of soccer-specific movements, including light ball-handling and agility drills.

The following instruments were used to conduct anthropometric measurements: Omron portable scale (Omron Healthcare Co., Kyoto, Japan) with a measurement accuracy of 0.5 kg; and caliper with a pressure of 10 g/mm^2^ at the ends; Martin anthropometer GPM 101 (GPM GmbH, Kloten, Switzerland) with a measurement accuracy of 1 mm; metal tape measure with a length of 150 cm; pelvimeter with a measurement accuracy of 1 mm; measuring stick with a measurement accuracy of 1 mm [18], as components of the Large Anthropometric Kit 113.

Length measurements of the body height, left arm and left leg were performed with an anthropometer.

The circumference of the thigh and calf was measured with a measuring tape. Diameters of the hips, the knee joint, and the ankle joint were measured with a pelvimeter. Skinfold thickness of the calf, abdomen, thigh, and back was measured with a caliper. Body mass was calculated with a weight scale [19]. All measurements were conducted by trained raters.

#### 2.3.1. Program and Procedure for Measuring Motor Skills

All manifest motor skills were measured following the methods of Kurelić and associates (1975) [20].

Trunk Lift for 30 s. The subject lies on their back with knees bent at a 90° angle. Hands are crossed on the chest, and a partner stabilizes the feet. The subject performs trunk lifts with a twist, alternating between left knee–right elbow and vice versa for 30 s.

Push-Ups on the Floor for 30 s. The subject lies on their stomach with arms bent at a 90° angle at the elbow joints. A correct push-up is one where the chest touches the floor, and the arms fully extend at the top of each repetition. The number of correct Push-Ups completed in 30 s is recorded.

Flexed-Arm Hang. The subject hangs on the bar with their chin above the bar. The measurement is taken while the subject tries to hold this position for as long as possible.

Standing Long Jump. Mat, springboard, metal tape measure. The subject, standing on a reversed springboard, jumps forward and lands on the mat. Three jumps are performed, and invalid attempts are repeated.

Trunk Flexion. Bench (40 cm high), wooden meter (60 cm), vertical positioning. The subject stands on the bench with legs straight and bends forward as far as possible without bending the knees or tilting the torso sideways. The depth of the reach in cm is recorded, with the better value from the two attempts taken.

Single-Leg Tapping for 20 s. Balance board with the ridge facing forward, chair without a backrest. The subject taps the board with one leg (chosen by the subject) from one side to the other for 20 s. Each tap and return to the starting position counts as one cycle. The total number of correctly performed cycles is recorded.

Single-Leg Balance with Closed Eyes. The subject stands on one leg with the other foot resting on the knee of the standing leg, arms on the hips. The test lasts a maximum of 60 s and is repeated 3 times. The total time (in seconds) from all three attempts is recorded (maximum of 180 s). Measurement starts when the subject closes their eyes. These procedures ensure a standardized approach to assessing motor skills, helping to maintain consistency and accuracy in the results.

#### 2.3.2. Program and Procedure for Measuring Specific Motor Skills

**Figure 2:**
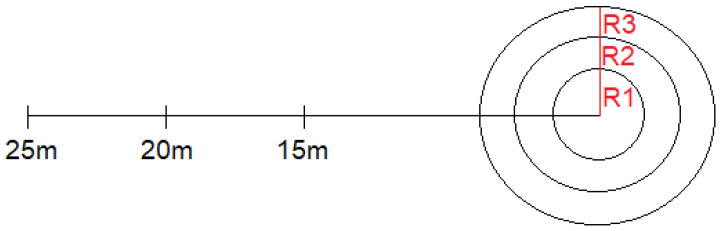
Horizontal Accuracy illustration.

For Horizontal Accuracy, we used a flat grassy surface marked with three concentric circles with radii, R1, R2, and R3, of 1, 3, and 5 m, respectively, and a clear white line from which shots are taken at a distance of 15, 20 and 25 m (Figure 2). The subject aims at one of the three circles. The starting points for the shots are marked with A. Nine shots are taken from point A, using the preferred foot and a stationary ball (ball size: 5; mass: 400–450 g; pressure: 58.6–107.6 kPa; circumference: 68–70 cm). A hit in the centre circle earns 5 points, the second circle 3 points, and the outer circle 1 point.

**Figure 3:**
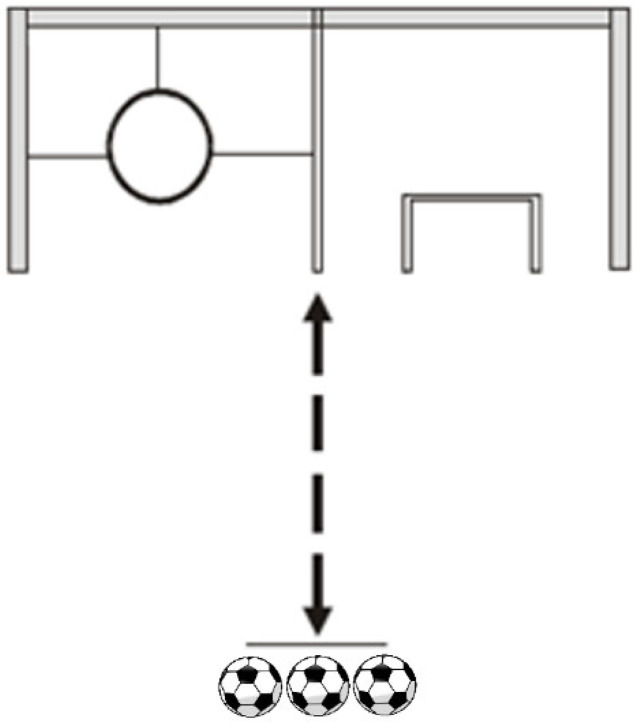
Vertical Accuracy illustration.

For Vertical Accuracy (Hoop and Goal), the subject kicks a ball so that it passes through a hoop. The distance from which the kicks are taken is 16 m (Figure 3). Each subject has three attempts. A ball that passes through the hoop earns 5 points; a ball that hits the hoop earns 3 points; and a ball that hits the square (a lowered target below the hoop) earns 1 point. A goal with dimensions of 120 cm long and 80 cm high is used, which is three standard footballs; a small goal (target) is placed on one half and on the goal line of the large goal (the large goal is divided in half by a target). The small goal is placed in the middle of the restricted area. Each subject must hit the small goal. Hitting the target (small goal) earns 5 points, hitting the post of the small goal earns 3 points, and hitting the post of the large goal or the target on the opposite side earns 1 point. Each subject gets three attempts, and the total score is recorded.

In the Single-Leg Ball Juggle and Alternating Ball Juggle, there is a smooth grass surface, a standard football, and a drawn circle with a radius of 3 m. The subject enters the circle and, with their hand, tosses the ball towards their preferred foot, trying to dribble it as fast as possible without going outside the circle. Two correct attempts are measured, and the best result is recorded. The test lasts 20 s.

**Figure 4:**
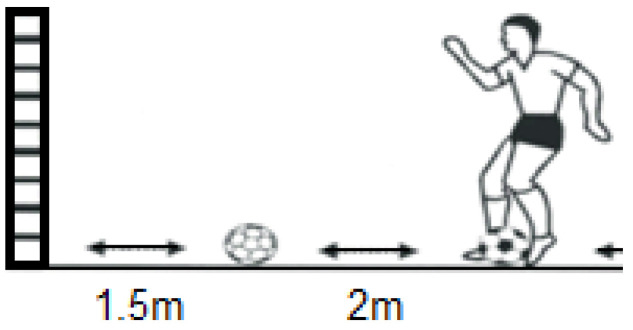
Wall Pass illustration.

In the Dominant Leg Wall Pass, Non-Dominant Leg Wall Pass, and Alternating Leg Wall Pass, the goal is to hit a ball against a wall and have it rebound past a specific line (Figure 4). The number of successful rebounds in 20 s is counted.

**Figure 5:**
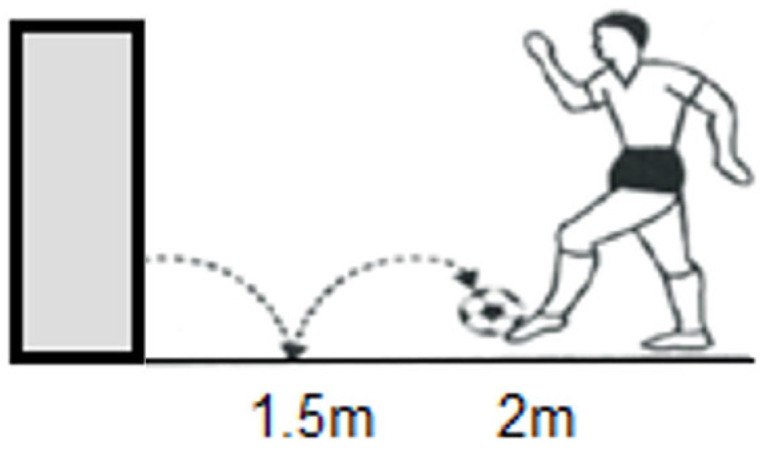
Parabolic Wall Pass illustration.

In the Dominant Leg Parabolic Wall Pass, Non-Dominant Leg Parabolic Wall Pass, and Alternating Leg Parabolic Wall Pass, the goal is to repeatedly bounce a ball off the ground and a wall, alternating between the two surfaces (Figure 5). The number of successful rebounds in 20 s is also counted.

In Sprint with Ball 20 m, Sprint with Ball 20 m Flying Start, and Sprint 20 m, the subject places the ball on the starting line and assumes a standing start position. Upon a visual signal, the subject starts and touches the ball once within the first 3 m and at least three more times in the remaining 17 m. Each subject gets two attempts, and the best time is recorded. Sprint with ball 20 m flying start is performed with the same procedure as sprint with ball 20 m. Sprint 20 m is performed without a ball.

**Figure 6:**
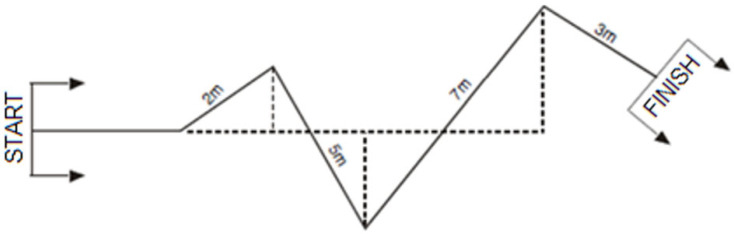
Slalom Test illustration.

In the Slalom Test with and without Ball, the participant dribbles the ball as quickly as possible around the flags, following the winding course (Figure 6). A successful attempt is completed when the participant crosses the finish line with the ball. Each subject gets two attempts, and the best time is recorded.

**Figure 7:**
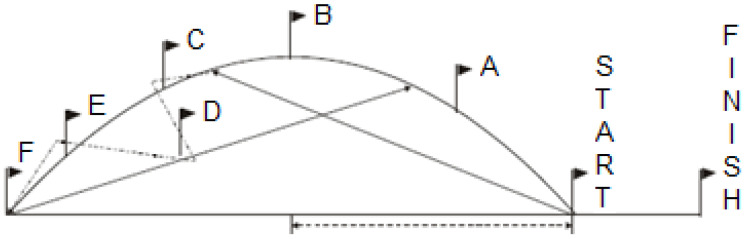
Semi-Circle Run illustration.

In Semi-Circle Run with and without Ball, upon a visual signal, the subject kicks the ball into the space between flags B and C and runs around the semi-circle as quickly as possible to meet the ball. After receiving the ball, the participant dribbles through flags C, D, and E. Upon reaching flag F, the participant kicks the ball back towards the space between flags B and A, and runs back around the semi-circle to meet the ball and dribble it through the finish line. The time is recorded from the moment the signal is given until the subject crosses the finish line with the ball (Figure 7). Each participant gets three attempts, and the best time is recorded.

**Figure 8:**
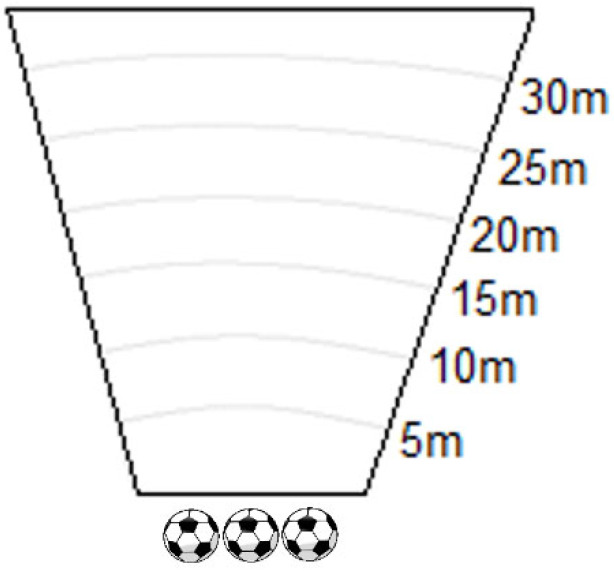
Explosive Power illustration.

In the Explosive Power of Dominant Leg, Non-Dominant Leg and Head testing, ball distance is measured. To measure the maximum force of a kick and the subsequent distance the ball travels, a flat, grassy field with marked lines every 5 m and three standard football balls is used (Figure 8). The subject takes three attempts at kicking the ball as hard as possible with the dominant leg, non-dominant leg and head. The best distance achieved is recorded.

### 2.4. Statistical Analysis

Statistical analyses were performed in SPSS statistical software (SPSS 23.0, IBM Inc., Chicago, IL, USA). All statistical tests were considered significant at *p* < 0.05.

The normality of the distributions for each variable was tested and confirmed using the Kolmogorov–Smirnov method. In the subsequent processing of the results, the interrelationship between the applied variables was examined for each subgroup separately, depending on each chronological age group, utilizing a correlation matrix. The factor structure for each chronological age group was determined using Exploratory Factor Analysis (EFA), which identifies latent dimensions by analyzing the shared variance among variables. The number of significant factors was determined using the Kaiser–Guttman criterion, followed by the application of Kaiser’s normal-varimax rotation to achieve an orthogonal solution. A varimax rotation was used to ensure distinct factor interpretability, reflecting the theoretical independence of the latent motor dimensions. Each extracted factor was interpreted based on the pattern of variable loadings (correlations between variables and factors). Prior to factor extraction, the suitability of the data was assessed using the Kaiser–Meyer–Olkin (KMO) measure of sampling adequacy and Bartlett’s test of sphericity. The significance of the factor loadings was determined using a threshold of 0.40. Finally, Supplementary Materials contain the results of the descriptive statistics presented in Tables S1–S21.

## 3. Results

**Figure 9:**
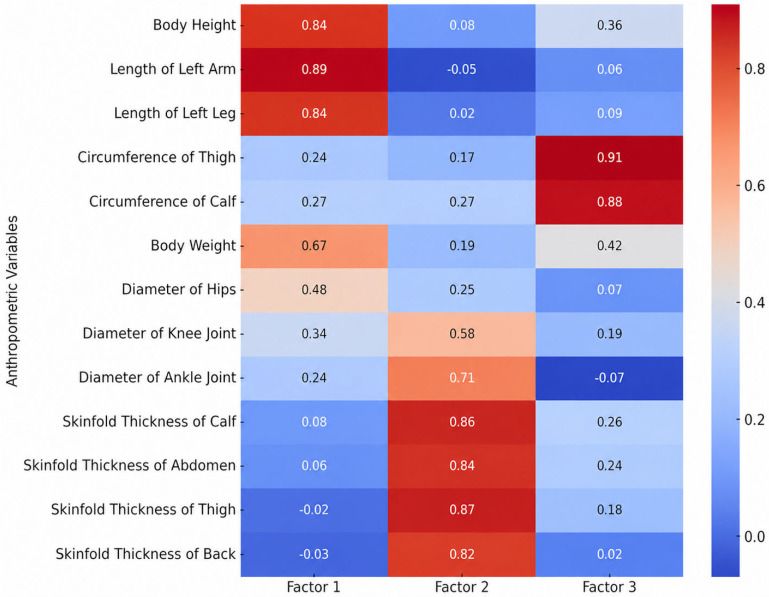
Factor loadings of anthropometric variables for the age group 11–13.

**Figure 10:**
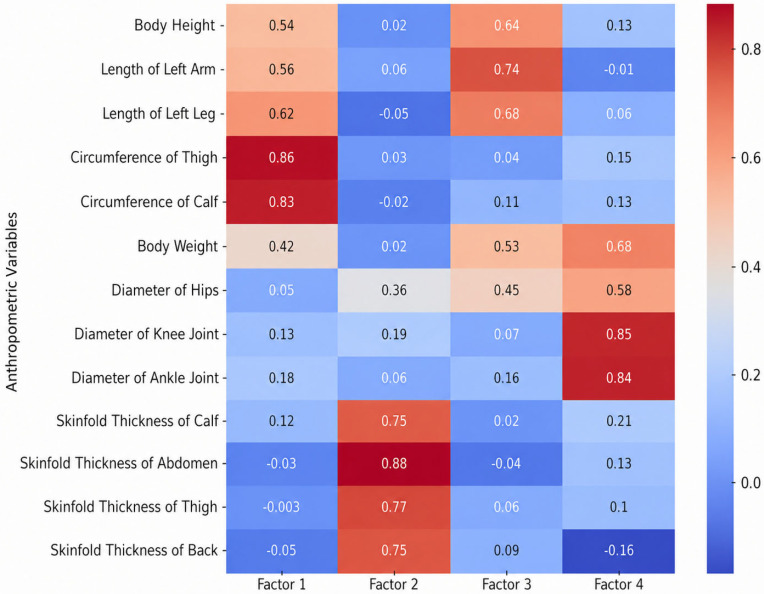
Factor loadings of anthropometric variables for the age group 13–15.

**Figure 11:**
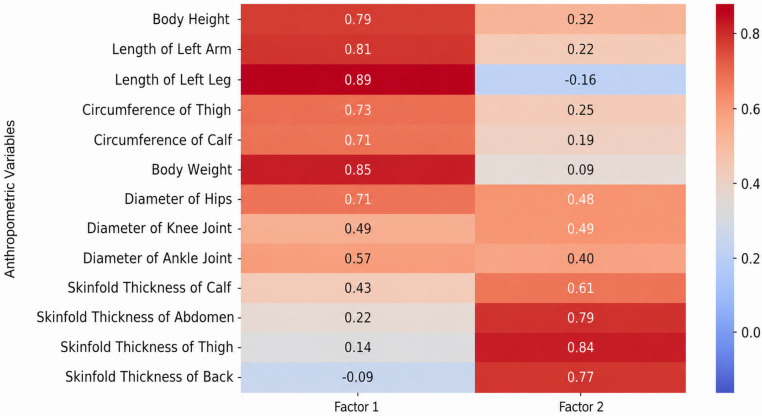
Factor loadings of anthropometric variables for the age group 15–17.

**Figure 12:**
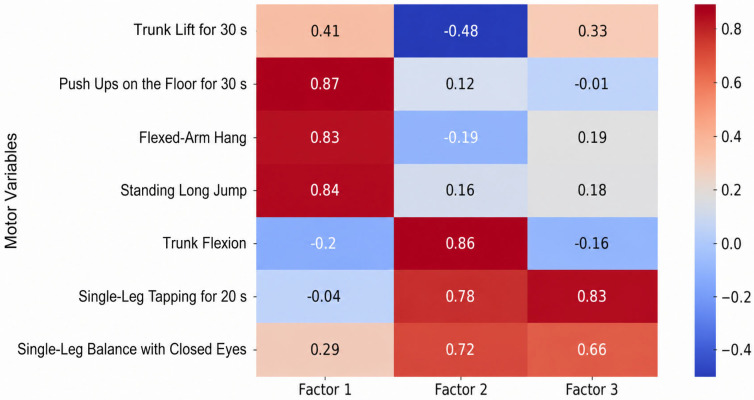
Factor loadings of motor variables for the age group 11–13.

**Figure 13:**
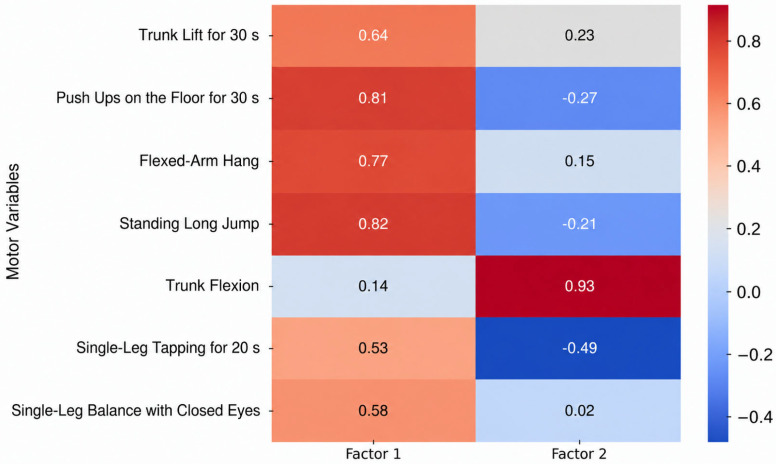
Factor loadings of motor variables for the age group 13–15.

**Figure 14:**
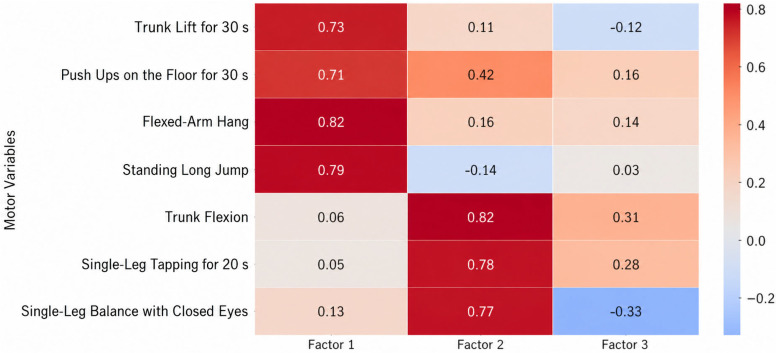
Factor loadings of motor variables for the age group 15–17.

**Figure 15:**
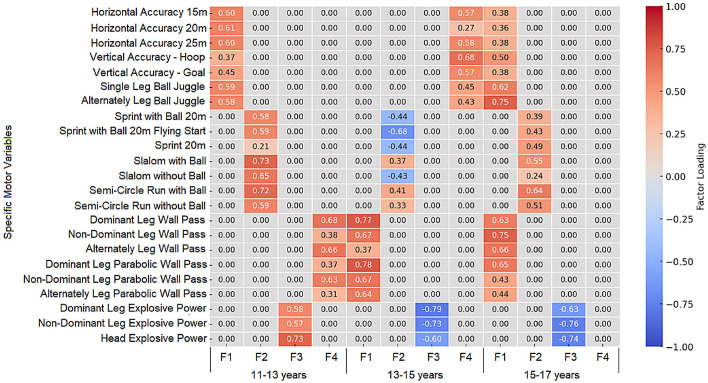
Factor loadings of specific motor skills for all age groups. Note: The value “0.00” represents no factor loading.

The adequacy of the sampling was verified via the Kaiser–Meyer–Olkin (KMO) measure, which ranged from 0.59 to 0.79 across all sets, and Bartlett’s test of sphericity, which reached statistical significance (*p* < 0.001) in all instances. The factor loadings of anthropometric characteristics, motor skills, and specific motor skills for the age groups 11–13, 13–15, and 15–17 are presented in Figure 9, Figure 10, Figure 11, Figure 12, Figure 13, Figure 14 and Figure 15. The color intensity indicates the strength of the loading of each variable on the factors.

For the 11–13 age group (Figure 9), the factor analysis identified a robust and well-distributed structure consisting of three primary dimensions, which collectively explained 66.1% of the total cumulative variance. The largest portion of the variance was accounted for by body mass and volume (Factor 2), with an Eigenvalue of 3.28 (contributing 25.2% of the total variance). This was closely followed by skeletal dimensions (Factor 1), which had an Eigenvalue of 3.19 and accounted for 24.5% of the variance. Finally, subcutaneous fat tissue (Factor 3) emerged as the third distinct component, explaining 2.12 of the variance (16.4%). This clear factorial differentiation appears to constitute a stable basis for evaluating the morphological characteristics of young players in this developmental phase.

The anthropometric structure for subjects aged 13–15 (Figure 10) proved to be more complex than in younger groups, shifting to a robust four-factor solution that explained 74.5% of the total cumulative variance. Subcutaneous fat tissue (Factor 2) emerged as a primary source of variance with an Eigenvalue of 2.67 (contributing 20.5% of the total variance). This was closely followed by body circumferences (Factor 1), which explained 20.5% (Eigenvalue = 2.66). The remaining dimensions were clearly differentiated into skeletal diameters (Factor 4), accounting for 18.3% (Eigenvalue = 2.38), and longitudinal skeletal dimensions (Factor 3), which contributed 15.1% (Eigenvalue = 1.96). This increased differentiation is potentially associated with the significant morphological changes occurring during the mid-adolescent growth spurt.

For the oldest group (15–17 years), the factor analysis revealed a more streamlined and integrated structure with two distinct factors (Figure 11), collectively explaining 64.4% of the total cumulative variance. The first dimension, General Morphological Status (Factor 1), emerged as the dominant factor with an Eigenvalue of 5.16, explaining 39.7% of the variance. This factor is primarily associated with body height, limb lengths, body weight, and circumferences, suggesting that skeletal dimensions and body mass dominate physical development in this age group. The second factor, Subcutaneous Fat Tissue (Factor 2), followed with an Eigenvalue of 3.21, explaining 24.7% of the variance, and was clearly defined by skinfold thickness measurements. This shift from a multi-factor structure in younger groups to a more consolidated two-factor model may indicate a higher level of morphological integration as subjects approach physical maturity.

The factor analysis for motor skills in subjects aged 11–13 (Figure 12) revealed a highly robust structure consisting of three distinct factors, which collectively explained 84.5% of the total cumulative variance. The first and most dominant factor, Factor 1 (Explosive and Repetitive Strength), had an Eigenvalue of 2.44 and accounted for 34.9% of the variance, with primary loadings from Push-Ups (0.87), Standing Long Jump (0.84), and flexed-arm Hang (0.83). The second dimension, Factor 2 (Flexibility and Coordination), explained 31.0% of the variance (Eigenvalue = 2.17), led by Trunk Flexion (0.86) and Single-Leg Tapping (0.78). The third factor, Factor 3 (Balance and Motor Control), contributed 18.6% to the variance (Eigenvalue = 1.30), primarily through Single-Leg Tapping (0.83) and Balance (0.66). This clear differentiation confirms that motor abilities in this age group are well-defined across strength, flexibility, and neuromuscular control.

For the 13–15 age group (Figure 13), the factor analysis of motor variables yielded a more integrated two-factor solution, collectively explaining 61.0% of the total cumulative variance. The first and most dominant dimension, General Motor Power and Stability (Factor 1), explained 42.6% of the variance (Eigenvalue = 2.98). This factor is characterized by high loadings from explosive and repetitive strength tasks (Push Ups: 0.81; Standing Long Jump: 0.82; Flexed-Arm Hang: 0.77), indicating that physical capacities related to the growth spurt and general athleticism are the primary drivers of motor performance in this period. The second dimension, Flexibility and Movement Speed (Factor 2), accounted for 18.4% of the variance (Eigenvalue = 1.29), primarily through Trunk Flexion (0.93). This transition to a simplified two-factor structure suggests that during peak maturation, general physical capacities dominate over more refined motor control, as specialized skills like coordination and flexibility become secondary to the rapid development of strength.

For the oldest age group (15–17 years), the factor analysis of motor variables (Figure 14) revealed three distinct factors that collectively explained 68.6% of the total cumulative variance. The dominant dimension, General Motor Skills (Factor 1), explained 33.7% of the variance (Eigenvalue = 2.36) with strong loadings from strength and endurance tasks (Flexed-Arm Hang: 0.82; Standing Long Jump: 0.79; Trunk Lift: 0.73). This indicates that fundamental physical capacities are strongly established by late adolescence. The second dimension, Specialized Motor Skills (Factor 2), accounted for 30.0% of the variance (Eigenvalue = 2.10) and reflects a higher level of movement regulation, specifically coordination and flexibility (Trunk Flexion: 0.82; Single-Leg Tapping: 0.78; Single-Leg Balance: 0.77). The third dimension, Situational Motor Skills (Factor 3), was the least influential, explaining 4.9% of the variance (Eigenvalue = 0.34). This structure indicates a more refined and stable motor profile, where primary physical capacities are consolidated, while specialized and situational skills represent distinct components of performance as players approach physical and technical maturity.

Football players (11–13 years)

The factor analysis of specific motor skills for the 11–13 age group (Figure 15) revealed a well-defined four-factor structure. The most significant component, Running Speed with and without a Ball (F2), achieved an Eigenvalue of 1.77, dominated by agility and slalom tasks (up to 0.729). The second factor, Intermuscular Coordination with a Ball (F1), followed with an Eigenvalue of 1.39, with significant loadings on horizontal accuracy and ball juggling tasks (up to 0.609). The remaining dimensions, Explosive Force (F3, Eigenvalue = 1.08) and Segmental Speed (F4, Eigenvalue = 1.04), effectively captured the variance in power and passing-related tasks. These four factors collectively represent the foundational technical structure for this age group. The model’s robustness is confirmed by communalities (h2) ranging from 0.51 to 0.84, indicating that the extracted factors provide a high-quality representation of the manifest technical motor space.

Football players (13–15 years)

For the 13–15 age group (Figure 15), the factor analysis identified a specialized four-factor structure. The primary dimension, Segmental Speed (F1), was the most robust with an Eigenvalue of 2.63, explaining a significant portion of the technical variance through wall-pass tasks (loadings up to 0.779). The remaining technical space was distributed among Running Speed (F2, Eigenvalue = 1.44), Explosive Force (F3, Eigenvalue = 1.53), and Intermuscular Coordination (F4, Eigenvalue = 1.91). Compared to the younger group, the 13–15 age group shows a higher degree of factor differentiation. This is expected in mid-adolescence as specific technical skills (like passing and explosive kicking) become more independent of each other due to the peak of the growth spurt.

Football players (15–17 years)

For the oldest age group (15–17 years, Figure 15), the specific motor space shows a higher degree of integration, reflecting professional-level technical consolidation. Although four factors were extracted, the primary dimension (F1—Technical Integration, Eigenvalue = 3.61) represents a merger of intermuscular coordination and segmental speed, with high loadings from both juggling (0.746) and wall-pass tasks (0.746). The second factor, Running Speed (F2, Eigenvalue = 1.34), integrated sprinting and slalom performance (up to 0.636), while the third factor, explosive force (F3, Eigenvalue = 1.62), remained a distinct dimension characterized by high negative loadings in kicking and heading power (up to −0.759). A fourth factor (F4) emerged but lacked a clear, independent affiliation, suggesting that at this developmental stage, technical skills act as a unified functional system. The model’s validity is supported by communalities (h2) ranging from 0.41 to 0.81, confirming that the factor structure effectively captures the specialized motor profile of senior youth players.

## 4. Discussion

This study investigated the structure of anthropometric characteristics, motor skills and specific motor skills in young football players, examining how these factors vary with chronological age. Our data revealed three, four and two factors defined by anthropometric characteristics for pre-pioneers, pioneers, and cadets, respectively. Moreover, three factors were determined for motor skills in pre-pioneers, two in pioneers and three in cadet age, respectively. Finally, four factors were determined for specific motor skills in pre-pioneers, four in pioneers and three in cadet age, respectively.

Anthropometric characteristics are fundamental determinants of physical performance in football players [21,22]. Body mass, body height and fat mass percentage are highly associated with sprint time [23]. Our results yielded a balanced distribution, suggesting that the three factors provide distinct and important contributions to explaining the underlying structure of anthropometric characteristics in the age group of pre-pioneers. Biomechanically, this stable structure provides a foundational mechanical base for the acquisition of basic movement patterns before the onset of rapid pubertal changes. This reflects the distinction between chronological age and biological maturation, as the foundational structure remains consistent despite individual maturational timing in early adolescence [24]. The presence of three factors likely reflects multiple dimensions of physical development, such as skeletal dimensions, body mass and volume, and subcutaneous fat tissue, each of which is crucial during the growth phase of early adolescence. Another study confirmed that the circumference of the waist and hip differ between children aged under 12 years and children aged 12–15 years old [25]. Furthermore, in the pioneer age group, there is the presence of four factors indicating a broader range of physical characteristics influencing body structure during this developmental period. This diversification of the latent structure aligns with the non-linear growth of skeletal and muscular systems during the circumpubertal growth spurt. Factor 1 may relate to general morphological features, while Factor 2 likely represents key aspects such as subcutaneous fat tissue. Factors 3 and 4 likely capture more specific elements like body volume, mass, and joint dimensions. The results suggest that body composition and physical development diversify further as adolescents progress through this age range. Moreover, Staśkiewicz-Bartecka and associates [26] showed that fat-free mass is significantly different between younger and older football players. In the cadet group (15–17 years), Factor 2, accounting for 24.7% of the variance, is primarily linked to skinfold thickness measurements, indicating that this factor represents subcutaneous fat tissue. This distinction between skeletal and fat tissue development reflects a critical phase of physical maturation in late adolescence, where growth in body size stabilizes, and the physical structure becomes more specialized for high-intensity athletic demands, but variations in body composition, such as fat distribution, become more pronounced. This aligns with the findings of Bernal-Orozco et al. [27], who demonstrated that as players mature and move toward professional levels, body mass and muscle mass percentages vary significantly, requiring specific body composition optimization.

Motor performance in young football players has an important role [28,29]. Furthermore, motor performance increases over an adolescent period [30]. Our data showed that the motor performance of subjects aged 11–13 exhibits a prominent pattern of covariation, which can be interpreted as a general factor (Factor 1), which included strength variables (Push-Ups, Flexed Arm Hang and Standing Long Jump), with secondary contributions from more specialized factors (Factors 2 and 3) likely relating to coordination and movement control. However, physical performance can be advantageous mostly in young football players under 14 years [31]. Additionally, results in the age group 13–15 suggest that motor performance is primarily influenced by a broad general factor (Factor 1), related to energy and strength, with a secondary influence from more specialized skills (Factor 2), reflecting increasing complexity and improvement of movement skills during this developmental period. This shift in the factor structure demonstrates the impact of the maturational peak, where the growth spurt disrupts previously stable motor patterns, leading to temporary factor differentiation. Consequently, during this unstable developmental phase, strengthening exercises reduce the risk of injuries among football players [32]. Moreover, incorporating balance training in the routine of young players can contribute to higher agility performance [33]. Regarding the motor performance in subjects aged 15–17, physical abilities are primarily determined, with growing importance placed on coordination and more specialized skills as adolescents near adulthood. The distribution of explained variance indicates a combination of foundational motor strength and skill improvement. This transition from a predominant general motor factor to a more diversified structure reflects the model of motor differentiation. As noted by Hirtz and Starosta [34], maturation entails a shift from generalized motor foundations toward specialized coordination patterns essential for elite performance. Elzner [35] found that effective performance in football tasks requiring agility, foot coordination, and precision largely depends on general motor ability. He identified two player types among young footballers: those with high explosive power and good muscle tone regulation, better suited for modern football, and those with developed static and repetitive muscle power, less aligned with the demands of the game.

Our findings on specific motor skills, including intermuscular coordination and segmental speed of the lower extremities with a ball, reveal age-related improvements that may be attributed to neuromuscular maturation. In the 11–13 years group, foundational movement patterns and basic dribbling skills are emphasized as coordination and speed are still in early development. In the 13–15-year-old group, rapid gains in neuromuscular refinement and physical growth enable more complex and precise movements. Our results indicate that the most significant gains often occur during this transition, which aligns with findings from Nowak et al. (2025) [36]. Their data on young footballers (aged 12–16) demonstrated that the most substantial improvements in sprint speed and lower-limb power occur between the ages of 13 and 14, mirroring the trends observed in our 13–15-year-old group. By 15–17 years, players demonstrate advanced motor skills, integrating speed and coordination into gameplay with a focus on precision and tactical decision-making under pressure. This observed motor specialization demonstrates that as players approach professional maturity, training models must transition toward high-intensity, game-like scenarios to optimize explosive force and resilience [37]. Similarly, running speed with and without a ball and explosive force in hitting a ball with the foot and head improve progressively across all cohorts. Cardoso et al. (2025) [38] demonstrate that significant seasonal increases in maximal theoretical speed are particularly evident in the under-15 category, highlighting the dynamic nature of sprinting performance during these maturational periods. While the youngest players demonstrate linear speed, basic control, and striking fundamentals, the 13–15-year-old group showed gains in power and accuracy, likely reflecting the non-linear development of physical qualities during the growth spurt [24]. In 15–17-year-olds, training emphasizes game-like, high-intensity scenarios, optimizing explosive force and speed for peak performance.

Football movements are inherently non-standardized and must be executed within confined space and time, often under challenging conditions and dynamic situations that arise during the game, particularly in the presence of opposing players [39]. One of the limitations of this study was the focus on static motor skill assessments (e.g., Push-Ups and Standing Long Jump), which might not fully capture motor skills crucial for football performance. Furthermore, while factor analysis provides a robust latent overview, it acts as an interpretative limitation as it may oversimplify the complex biological interactions between training age and biological maturation.

The development of football has made significant progress in the areas of physical conditioning and general motor skills [30]. Experts in fields such as medicine have determined the influence of both internal and external factors, including physical activities like football, on human anthropometric characteristics [8]. Systematic and consistent training, in particular, can significantly impact various aspects of a football player’s morphology [40]. However, while the use of factor analysis was appropriate for identifying structures in the data, relying solely on extracted factors to explain physical development might oversimplify complex biological processes. Furthermore, these latent structures are likely influenced by biological maturation, training experience, and the relative age effect, which should be considered when interpreting the non-linear development of young players. The emergence of age-specific factor structures carries both theoretical and practical implications. Theoretically, it indicates that physical development in youth soccer is non-linear and requires age-distinct modeling of performance. Practically, these findings can guide coaches in tailoring training programs, prioritizing general motor foundations in younger cohorts while focusing on integrated, specialized skill automation as players approach the cadet level. Our findings offer key insights for optimizing youth football training by focusing on age-specific motor skills. In the pre-pioneer phase, strength and coordination should be prioritized; in the pioneer phase, focus should be on agility and tactical drills; and in the cadet phase, precision, decision-making, and high-intensity simulations to prepare players for competitive play should be emphasized. This age-related progression is consistent with the findings of Benitez-Sillero et al. [41], who demonstrated that performance variables significantly improve and differentiate as players progress through formative categories.

These strategies can guide training, injury prevention, and overall player development. Furthermore, this study did not account for positional roles, which represent a significant factor in the specialization of motor abilities as players mature. Future longitudinal or cross-sectional studies should explore how these latent dimensions further differentiate based on the specific tactical demands of different playing positions. Finally, the limitation of our study is the lack of determination of the biological maturity status of participants, which could have influenced the classification of players into the age groups (11–13, 13–15, and 15–17). While chronological age is commonly used in youth sport studies, biological maturity can vary significantly among individuals within the same age group and may impact physical performance [42].

## 5. Conclusions

This study revealed that the latent structure of the examined space is non-uniform, exhibiting structural variability across different age groups. Across all age groups, two consistent latent dimensions were identified in the anthropometric data: general morphological factors and subcutaneous fat tissue. In motor skills, an initial general factor was further divided into central and energetic regulation of movements. These findings were consistent across the age groups, although the overall dimensionality fluctuated between two and four factors, especially within specific motor skills, where a highly differentiated four-factor structure was observed in younger cohorts. In motor skills, the initial structure observed in the youngest group progressively differentiated into more specialized factors for the older cohorts.

In the pre-pioneers (11–13 years), the emphasis was on basic motor skills, with central and energetic regulation playing a significant role. In the pioneers (13–15 years), the separation of motor skills into more specialized components, such as intermuscular coordination and running speed with and without the ball, was more apparent, reflecting the refinement of motor control during this developmental phase. In the cadets (15–17 years), the factors related to segmental speed of the lower extremities and explosive force in hitting a ball with the foot and head gained prominence, reflecting a process of technical integration where previously independent skills become more functionally unified. These findings contribute to understanding football player development and help improve player selection and training processes.

## Data Availability

The data presented in this study are openly available (https://repository.ukim.mk/).

## Supplementary Materials

The following supporting information can be downloaded at: https://www.mdpi.com/article/10.3390/jfmk11020177/s1.
