# Supplementary material for: The Latent Dimensionality of Physical and Technical Performance Across Three Youth Soccer Tiers
Source: J Funct Morphol Kinesiol. 2026 Apr 28;11(2):177. doi: 10.3390/jfmk11020177 (PMC13214771; doi:10.3390/jfmk11020177)
Supplement: Supplementary file 1 [file jfmk-11-00177-s001.zip › jfmk-4279593-supplementary.pdf]

Supplementary material

Table S1. Descriptive statistics of anthropometric variables for the age group 11-13

Variable	Valid N	Mean	Min.	Max.	Std.Dev.	Skju.	Kurt.
**Body Height**	133	1589.25	1480.00	1720.00	59.36	.15	-.76
**Length of left arm**	133	728.87	590.00	830.00	47.68	-.11	-.61
**Length of left leg**	133	895.56	720.00	1030.00	65.44	-.18	-.37
**Circumference of thigh**	133	355.94	250.00	570.00	66.08	.65	.21
**Circumference of calf**	133	246.09	140.00	420.00	56.70	.67	-.27
**Body weight**	133	46.61	32.00	61.00	59.20	0.54	0.23
**Diameter of hips**	133	256.84	220.00	290.00	12.54	-.32	.94
**Diameter of knee joint**	133	79.41	40.00	85.00	59.62	.93	-.43
**Diameter of ankle joint**	133	58.87	40.00	90.00	8.93	.66	.99
**Skinfold thickness of calf**	133	6.21	4.00	9.00	1.17	.37	-.46
**Skinfold thickness of abdomen**	133	5.71	4.00	8.00	.94	.12	-.19
**Skinfold thickness of thigh**	133	5.34	3.00	8.00	.99	.43	.10
**Skinfold thickness of back**	133	4.59	3.00	7.00	.84	.50	.29

Table S2. Descriptive statistics of anthropometric variables for the age group 13-15

Variable	Valid N	Mean	Min.	Max.	Std.Dev.	Skju.	Kurt.
**Body Height**	160	1699.56	1500.00	1790.00	52.53	-1.38	2.78
**Length of left arm**	160	787.88	680.00	990.00	53.33	.70	1.77
**Length of left leg**	160	1047.19	893.00	1233.00	92.27	1.66	2.50
**Circumference of thigh**	160	500.00	360.00	653.00	73.86	1.49	2.76
**Circumference of calf**	160	353.69	190.00	520.00	62.28	.04	.72
**Body weight**	160	55.50	30.00	70.00	68.56	-.63	1.13
**Diameter of hips**	160	266.94	240.00	290.00	15.17	-.11	-1.02
**Diameter of knee joint**	160	71.66	50.00	90.00	10.21	-.07	-.49
**Diameter of ankle joint**	160	60.12	40.00	80.00	9.14	.26	-.03
**Skinfold thickness of calf**	160	6.04	4.00	10.00	.91	.86	3.66
**Skinfold thickness of abdomen**	160	5.52	3.00	9.00	.93	.91	1.99
**Skinfold thickness of thigh**	160	5.20	4.00	8.00	.83	.61	.70
**Skinfold thickness of back**	160	4.54	3.00	7.00	.80	.93	.88

Table S3. Descriptive statistics of anthropometric variables for the age group 15-17

Variable	Valid N	Mean	Min.	Max.	Std.Dev.	Skju.	Kurt.
**Body Height**	134	1746.27	1660.00	1860.00	44.96	.081	-.69
**Length of left arm**	134	835.37	80.00	930.00	75.03	-.75	1.88
**Length of left leg**	134	1283.43	920.00	1670.00	106.57	1.80	2.50
**Circumference of thigh**	134	601.57	440.00	789.00	598.91	1.06	2.27
**Circumference of calf**	134	392.01	310.00	500.00	31.52	-.15	1.01

**Body weight**	134	62.31	61.00	79.00	8.80	-2.12	1.18
**Diameter of hips**	134	279.48	250.00	310.00	12.99	.28	.00
**Diameter of knee joint**	134	78.88	50.00	110.00	9.82	.14	1.00
**Diameter of ankle joint**	134	65.99	50.00	80.00	12.06	-2.12	1.90
**Skinfold thickness of calf**	134	6.95	4.00	12.00	1.26	.55	1.51
**Skinfold thickness of abdomen**	134	6.23	4.00	10.00	1.28	.20	-.42
**Skinfold thickness of thigh**	134	5.58	4.00	11.00	1.14	.96	2.23
**Skinfold thickness of back**	134	5.06	4.00	11.00	1.04	1.55	1.33

Table S4. Descriptive statistics of motoric variables for the age group 11-13

Variable	Valid N	Mean	Min.	Max.	Std.Dev.	Skju.	Kurt.
**Trunk lift for 30 s**	133	18.83	9.00	34.00	3.68	.30	1.56
**Push Ups on the floor for 30 s**	133	6.06	0.00	19.00	3.71	.98	.80
**Flexed-Arm Hang**	133	39.39	17.00	85.00	12.51	.93	1.77
**Standing Long jump**	133	172.79	108.00	221.00	19.82	.36	.81
**Trunk Flexion**	133	33.80	24.00	54.00	21.00	.64	-.26
**Single-Leg Tapping for 20 s**	133	28.91	21.00	33.00	3.27	1.81	2.52
**Single-Leg Balance with Closed Eyes**	133	100.82	68.00	130.00	12.42	.09	.09

Table S5. Descriptive statistics of motoric variables for the age group 13-15

Variable	Valid N	Mean	Min.	Max.	Std.Dev.	Skju.	Kurt.
**Trunk lift for 30 s**	160	21.125	11.00	37.00	5.39	.57	-.05
**Push Ups on the floor for 30 s**	160	10.150	0.00	25.00	5.55	.24	-.86
**Flexed-Arm Hang**	160	48.912	17.00	96.00	16.73	.63	.06
**Standing Long jump**	160	205.95	181.00	281.00	35.28	-1.77	4.79
**Trunk Flexion**	160	43.931	34.00	61.00	21.46	.87	-.19
**Single-Leg Tapping for 20 s**	160	29.744	22.00	37.00	3.03	.28	.19
**Single-Leg Balance with Closed Eyes**	160	102.575	61.00	161.00	18.52	.96	.69

Table S6. Descriptive statistics of motoric variables for the age group 15-17

Variable	Valid N	Mean	Min.	Max.	Std.Dev.	Skju.	Kurt.
**Trunk lift for 30 s**	134	23.69	12.00	31.00	3.03	-1.45	3.20
**Push Ups on the floor for 30 s**	134	11.84	4.00	19.00	3.11	-.19	-.45
**Flexed-Arm Hang**	134	56.97	34.00	85.00	9.36	.12	-.07
**Standing Long jump**	134	243.38	155.00	269.00	19.76	-2.24	4.92
**Trunk Flexion**	134	51.71	42.00	66.00	21.88	1.11	4.54
**Single-Leg Tapping for 20 s**	134	31.06	24.0	38.00	3.22	-.05	-.69
**Single-Leg Balance with Closed Eyes**	134	115.12	95.00	144.00	14.12	.35	-1.17

Table S7. Descriptive statistics of specific motor skills for the age group 11-13

Variable	Valid N	Mean	Min.	Max.	Std.Dev.	Skju.	Kurt.
**Horizontal Accuracy 15m**	133	7.89	3.00	15.00	2.30	.06	-.08
**Horizontal Accuracy 20m**	133	6.65	1.00	11.00	2.19	-.20	.02
**Horizontal Accuracy 25m**	133	4.15	0.00	9.00	2.08	-.13	-.45
**Vertical Accuracy - Hoop**	133	3.18	0.00	9.00	2.17	.24	-.79
**Vertical Accuracy - Goal**	133	2.53	0.00	9.00	2.13	.93	.40
**Single Leg Ball Juggle**	133	16.82	4.00	26.00	4.02	.00	.05
**Alternately Leg Ball Juggle**	133	12.90	4.00	26.00	4.78	.64	-.24
**Dominant Leg Wall Pass**	133	8.52	3.00	16.00	2.68	-.01	-.29
**Non-Dominant Leg Wall Pass**	133	6.40	2.00	11.00	2.14	-.06	-.72
**Alternately Leg Wall Pass**	133	7.03	3.00	12.00	2.11	.23	-.40
**Dominant Leg Parabolic Wall Pass**	133	5.42	1.00	9.00	1.54	-.33	.28
**Non-Dominant Leg Parabolic Wall Pass**	133	4.41	2.00	9.00	1.51	.38	-.25
**Alternately Leg Parabolic Wall Pass**	133	5.39	2.00	9.00	1.52	.11	-.37
**Sprint with Ball 20m**	133	4.39	3.50	5.20	.40	.01	-1.14
**Sprint with Ball 20m Flying Start**	133	4.02	3.35	4.92	.33	.55	.09
**Sprint 20m**	133	4.87	4.00	5.80	.43	-.23	-.47
**Slalom with Ball**	133	6.38	4.15	8.10	.71	-.79	1.94
**Slalom without Ball**	133	4.82	3.30	6.90	.94	.28	-1.35
**Semi-Circle Run with Ball**	133	20.92	12.75	34.73	4.3	.69	-.27
**Semi-Circle Run without Ball**	133	14.47	10.70	19.70	1.53	.70	1.45
**Dominant Leg Explosive Power**	133	24.82	13.00	36.00	1.19	.25	-.03
**Non-Dominant Leg Explosive Power**	133	12.76	6.00	24.00	3.33	.94	.96
**Head Explosive Power**	133	5.23	5.00	15.00	2.18	6.15	41.82

Table S8. Descriptive statistics of specific motor skills for the age group 13-15

Variable	Valid N	Mean	Min.	Max.	Std.Dev.	Skju.	Kurtosis
**Horizontal Accuracy 15m**	160	8.88	3.00	15.00	2.79	.34	-.56
**Horizontal Accuracy 20m**	160	7.13	0.00	11.00	2.09	-.20	.29
**Horizontal Accuracy 25m**	159	4.68	0.00	11.00	2.29	-.14	-.26
**Vertical Accuracy - Hoop**	160	3.36	0.00	9.00	2.51	.301	-.72
**Vertical Accuracy - Goal**	160	2.94	0.00	25.00	3.08	3.51	2.06
**Single Leg Ball Juggle**	160	18.51	1.00	93.00	9.60	5.83	4.33
**Alternately Leg Ball Juggle**	160	14.73	3.00	72.00	8.35	4.21	2.24
**Dominant Leg Wall Pass**	160	9.84	3.00	21.00	3.30	.29	.21
**Non-Dominant Leg Wall Pass**	160	6.57	2.00	14.00	2.22	.52	.11
**Alternately Leg Wall Pass**	160	8.08	3.00	16.00	2.84	.55	-.30
**Dominant Leg Parabolic Wall Pass**	160	5.55	2.00	12.00	1.78	.43	.36
**Non-Dominant Leg Parabolic Wall Pass**	160	4.18	1.00	8.00	1.50	.36	-.44

**Alternately Leg Parabolic Wall Pass**	160	5.33	1.00	9.00	1.47	.27	.36
**Sprint with Ball 20m**	160	3.51	2.90	5.00	.39	.86	1.14
**Sprint with Ball 20m Flying Start**	160	3.19	2.30	5.00	.48	2.09	5.52
**Sprint 20m**	160	4.28	3.00	10.40	.78	5.94	4.61
**Slalom with Ball**	160	6.93	5.66	10.00	.78	1.28	1.83
**Slalom without Ball**	160	4.18	3.28	5.60	.44	.45	-.17
**Semi-Circle Run with Ball**	160	21.54	20.00	31.07	1.34	4.69	3.49
**Semi-Circle Run without Ball**	160	18.75	16.19	20.25	.72	-.44	.19
**Dominant Leg Explosive Power**	160	27.38	19.00	41.00	6.22	.60	.11
**Non-Dominant Leg Explosive Power**	160	14.25	9.00	28.00	4.61	1.13	.28
**Head Explosive Power**	160	6.00	8.00	19.00	2.55	1.32	3.83

Table S9. Descriptive statistics of specific motor skills for the age group 15-17

Variable	Valid N	Mean	Min.	Max.	Std.Dev.	Skju.	Kurt.
**Horizontal Accuracy 15m**	134	9.49	1.00	15.00	2.30	-.55	.90
**Horizontal Accuracy 20m**	134	6.49	3.00	13.00	1.93	.70	1.14
**Horizontal Accuracy 25m**	134	5.04	1.00	9.00	1.54	.62	-.39
**Vertical Accuracy - Hoop**	134	3.84	0.00	8.00	1.61	.03	.45
**Vertical Accuracy - Goal**	134	3.13	0.00	7.00	1.46	.29	-.01
**Single Leg Ball Juggle**	134	19.54	10.00	35.00	4.23	.05	.37
**Alternately Leg Ball Juggle**	134	13.85	4.00	27.00	5.42	.65	-.33
**Dominant Leg Wall Pass**	134	8.87	4.00	15.00	1.67	-.09	1.85
**Non-Dominant Leg Wall Pass**	134	7.05	4.00	12.00	1.56	-.09	.29
**Alternately Leg Wall Pass**	134	6.86	4.00	12.00	1.76	.34	-.40
**Dominant Leg Parabolic Wall Pass**	134	5.90	3.00	10.00	1.34	.46	.20
**Non-Dominant Leg Parabolic Wall Pass**	134	4.80	3.00	8.00	1.08	.69	.71
**Alternately Leg Parabolic Wall Pass**	134	5.52	3.00	9.00	1.09	-.21	.55
**Sprint with Ball 20m**	134	4.42	3.25	10.00	8.41	11.55	1.69
**Sprint with Ball 20m Flying Start**	134	3.26	2.87	3.90	.16	.94	1.63
**Sprint 20m**	134	4.05	3.51	4.87	.24	1.63	2.82
**Slalom with Ball**	134	6.07	3.65	6.88	.54	-1.61	4.81
**Slalom without Ball**	134	4.00	3.25	4.91	.39	.16	-.89
**Semi-Circle Run with Ball**	134	20.26	17.15	22.30	.84	-.61	2.28
**Semi-Circle Run without Ball**	134	8.02	10.00	16.00	7.24	1.46	1.27
**Dominant Leg Explosive Power**	134	29.51	22.00	39.00	4.53	.37	-.73
**Non-Dominant Leg Explosive Power**	134	16.22	6.00	30.00	5.1	.40	-.87
**Head Explosive Power**	134	7.25	10.00	14.00	2.73	.20	-.29

Table S10. Bartlett's Test of Sphericity and KMO - Anthropometric

11-13			13-15			15-17		
χ^2	df	p	χ^2	df	p	χ^2	df	p
820	78	<.001	192	21	<.001	250	21	<.001
KMO overall = 0.79			KMO overall = 0.69			KMO overall = 0.75		

Table S11. Bartlett's Test of Sphericity and KMO – Motor skills

11-13			13-15			15-17		
χ^2	df	p	χ^2	df	p	χ^2	df	p
217	21	<.001	313	21	<.001	219	21	<.001
KMO overall = 0.74			KMO overall = 0.69			KMO overall = 0.69		

Table S12. Bartlett's Test of Sphericity and KMO – Specific motor skills

11-13			13-15			15-17		
χ^2	df	p	χ^2	df	p	χ^2	df	p
233	21	<.001	1155	231	<.001	837	253	<.001
KMO overall = 0.64			KMO overall = 0.59			KMO overall = 0.63		

Table S13. Factor loadings of anthropometric variables for the age group 11-13.

Variable	Factor 1	Factor 2	Factor 3	Communalities (h2)
**Body Height**	0.84	0.07	0.36	0.77
**Length of left arm**	0.88	-0.05	0.06	0.70
**Length of left leg**	0.84	0.02	0.09	0.58
**Circumference of thigh**	0.24	0.17	0.91	0.81
**Circumference of calf**	0.27	0.27	0.88	0.84
**Body weight**	0.67	0.19	0.42	0.58
**Diameter of hips**	0.48	0.25	0.07	0.24
**Diameter of knee joint**	0.34	0.57	0.20	0.51
**Diameter of ankle joint**	0.24	0.70	-0.07	0.44
**Skinfold thickness of calf**	0.08	0.86	0.25	0.55
**Skinfold thickness of abdomen**	0.06	0.84	0.24	0.52
**Skinfold thickness of thigh**	-0.02	0.87	0.18	0.47
**Skinfold thickness of back**	-0.03	0.82	0.02	0.41
% of Variance	38.78	18.53	8.75	
Cumul. %	38.78	57.31	66.06	
Expl. Var	3.18	3.28	2.12	

Table S14. Factor loadings of anthropometric variables for the age group 13-15.

Variable	Factor 1	Factors 2	Factor 3	Factor 4	Communalities (h2)
**Body Height**	0.54	0.02	0.64	0.13	0.57
**Length of left arm**	0.56	0.06	0.74	-0.01	0.44
**Length of left leg**	0.62	-0.05	0.68	0.06	0.12
**Circumference of thigh**	0.86	0.03	0.04	0.15	0.53
**Circumference of calf**	0.83	-0.02	0.10	0.13	0.48
**Body weight**	0.42	0.02	0.53	0.68	0.48
**Diameter of hips**	0.05	0.36	0.45	0.58	0.33
**Diameter of knee joint**	0.13	0.19	0.07	0.85	0.43
**Diameter of ankle joint**	0.18	0.03	0.16	0.84	0.42
**Skinfold thickness of calf**	0.12	0.75	0.01	0.21	0.52
**Skinfold thickness of abdomen**	-0.03	0.87	-0.04	0.13	0.65
**Skinfold thickness of thigh**	0.00	0.77	0.06	0.10	0.54
**Skinfold thickness of back**	-0.05	0.75	0.09	-0.16	0.41
% of Variance	27.72	18.86	10.11	8.64	
Cumul. %	27.72	46.58	56.69	65.33	
Expl. Var	2.13	2.65	1.96		

Table S15. Factor loadings of anthropometric variables for the age group 15-17.

Variable	Factor 1	Factors 2	Communalities (h2)
**Body Height**	0.79	0.32	.793442
**Length of left arm**	0.81	0.22	.731426
**Length of left leg**	0.89	-0.16	.769407
**Circumference of thigh**	0.73	0.25	.615575
**Circumference of calf**	0.71	0.19	.629271
**Body weight**	0.85	0.09	.768849
**Diameter of hips**	0.71	0.48	.719048
**Diameter of knee joint**	0.49	0.49	.771160
**Diameter of ankle joint**	0.57	0.4	.776200
**Skinfold thickness of calf**	0.43	0.61	.601561
**Skinfold thickness of abdomen**	0.22	0.79	.600573
**Skinfold thickness of thigh**	0.14	0.84	.621039
**Skinfold thickness of back**	-0.09	0.77	.460090
% of Variance	50.42	15.47	
Cumul. %	50.42	65.9	
Expl. Var	5.19	3.38	

Table S16. Factor loadings of motor skills variables for the age group 11-13.

Variable	Factor 1	Factors2	Factor 3	Communalities (h2)
**Trunk lift for 30 s**	0.41	-0.48	0.33	.16
**Push Ups on the floor for 30 s**	0.87	0.12	-0.01	.530
**Flexed-Arm Hang**	0.83	-0.19	0.19	.54
**Standing Long jump**	0.84	0.16	0.18	.53
**Trunk Flexion**	-0.20	-0.85	-0.16	.12
**Single-Leg Tapping for 20 s**	-0.04	0.78	0.83	.07
**Single-Leg Balance with Closed Eyes**	0.29	0.72	0.66	.17
% of Variance	38.75	15.84	14.82	
Cumul. %	38.75	54.59	69.41	
Expl. Var	2.43	1.09	1.33	

Table S17. Factor loadings of motor skills variables for the age group 13-15.

Variable	Factor 1	Factors2	Communalities (h2)
**Trunk lift for 30 s**	0.64	0.23	0.26
**Push Ups on the floor for 30 s**	0.81	-0.27	0.65
**Flexed-Arm Hang**	0.77	0.15	0.51
**Standing Long jump**	0.82	-0.21	0.57
**Trunk Flexion**	0.14	0.93	0.31
**Single-Leg Tapping for 20 s**	0.53	-0.49	0.29
**Single-Leg Balance with Closed Eyes**	0.58	0.02	0.22
% of Variance	43.07	18.09	
Cumul. %	43.07	61.16	
Expl. Var	2.97	1.3	

Table S18. Factor loadings of motor skills variables for the age group 15-17.

Variable	Factor 1	Factors2	Factor 3	Communalities (h2)
**Trunk lift for 30 s**	0.73	0.11	-0.12	0.34
**Push Ups on the floor for 30 s**	0.71	0.42	0.16	0.56
**Flexed-Arm Hang**	0.82	0.16	0.14	0.55
**Standing Long jump**	0.79	-0.14	0.03	0.33
**Trunk Flexion**	0.06	0.02	0.91	0.05
**Single-Leg Tapping for 20 s**	0.05	0.78	0.28	0.19
**Single-Leg Balance with Closed Eyes**	0.13	0.78	-0.33	0.22
% of Variance	37.85	17.16	14.98	
Cumul. %	37.85	55.01	70.00	
Expl. Var	2.37	1.45		

Table S19. Factor loadings of specific motor skills variables for the age group 11-13.

Variable	Factor 1	Factor 2	Factor 3	Factor 4	Communalities (h2)
**Horizontal Accuracy 15m**	0.60	0.00	0.00	0.00	0.59
**Horizontal Accuracy 20m**	0.61	0.00	0.00	0.00	0.55
**Horizontal Accuracy 25m**	0.60	0.00	0.00	0.00	0.42
**Vertical Accuracy - Hoop**	0.37	0.00	0.00	0.00	0.39
**Vertical Accuracy - Goal**	0.45	0.00	0.00	0.00	0.34
**Single Leg Ball Juggle**	0.59	0.00	0.00	0.00	0.64
**Alternately Leg Ball Juggle**	0.58	0.00	0.00	0.00	0.59
**Sprint with Ball 20m**	0.00	0.58	0.00	0.00	0.80
**Sprint with Ball 20m Flying Start**	0.00	0.59	0.00	0.00	0.70
**Sprint 20m**	0.00	0.21	0.00	0.00	0.43
**Slalom with Ball**	0.00	0.12	0.00	0.00	0.46
**Slalom without Ball**	0.00	0.73	0.00	0.00	0.51
**Semi-Circle Run with Ball**	0.00	0.65	0.00	0.00	0.44
**Semi-Circle Run without Ball**	0.00	0.72	0.00	0.00	0.72
**Dominant Leg Wall Pass**	0.00	0.59	0.00	0.68	0.65
**Non-Dominant Leg Wall Pass**	0.00	0.00	0.00	0.38	0.73
**Alternately Leg Wall Pass**	0.00	0.00	0.00	0.66	0.50
**Dominant Leg Parabolic Wall Pass**	0.00	0.00	0.00	0.37	0.59
**Non-Dominant Leg Parabolic Wall Pass**	0.00	0.00	0.00	0.63	0.55
**Alternately Leg Parabolic Wall Pass**	0.00	0.00	0.00	0.31	0.49
**Dominant Leg Explosive Power**	0.00	0.00	0.58	0.00	0.45

**Non-Dominant Leg Explosive Power**	0.00	0.00	0.57	0.00	0.45
**Head Explosive Power**	0.00	0.00	0.73	0.00	0.46
% of Variance	22.94	13.50	7.81	6.57	
Cumul. %	22.94	36.44	44.25	50.82	
Expl. Var	4.03	3.54	2.94	3.21	

Note: The values "0.00" represent no factor loading.

Table S20. Factor loadings of specific motor skills variables for the age group 13-15.

**Variable**	**Factor 1**	**Factor 2**	**Factor 3**	**Factor 4**	**Communalities (h2)**
**Horizontal Accuracy 15m**	0.00	0.00	0.00	0.57	0.55
**Horizontal Accuracy 20m**	0.00	0.00	0.00	0.27	0.30
**Horizontal Accuracy 25m**	0.00	0.00	0.00	0.58	0.54
**Vertical Accuracy - Hoop**	0.00	0.00	0.00	0.69	0.46
**Vertical Accuracy - Goal**	0.00	0.00	0.00	0.58	0.27
**Single Leg Ball Juggle**	0.00	0.00	0.00	0.45	0.56
**Alternately Leg Ball Juggle**	0.00	0.00	0.00	0.43	0.44
**Sprint with Ball 20m**	0.00	-0.44	0.00	0.00	0.63
**Sprint with Ball 20m Flying Start**	0.00	-0.68	0.00	0.00	0.55
**Sprint 20m**	0.00	-0.44	0.00	0.00	0.66
**Slalom with Ball**	0.00	0.37	0.00	0.00	0.62
**Slalom without Ball**	0.00	-0.43	0.00	0.00	0.54
**Semi-Circle Run with Ball**	0.00	0.41	0.00	0.00	0.55
**Semi-Circle Run without Ball**	0.00	0.33	0.00	0.00	0.51
**Dominant Leg Wall Pass**	0.77	0.00	0.00	0.00	0.55
**Non-Dominant Leg Wall Pass**	0.67	0.00	0.00	0.00	0.30
**Alternately Leg Wall Pass**	0.37	0.00	0.00	0.00	0.45
**Dominant Leg Parabolic Wall Pass**	0.78	0.00	0.00	0.00	0.26
**Non-Dominant Leg Parabolic Wall Pass**	0.67	0.00	0.00	0.00	0.30
**Alternately Leg Parabolic Wall Pass**	0.64	0.00	0.00	0.00	0.32
**Dominant Leg Explosive Power**	0.00	0.00	-0.79	0.00	0.58
**Non-Dominant Leg Explosive Power**	0.00	0.00	-0.73	0.00	0.47
**Head Explosive Power**	0.00	0.00	-0.60	0.00	0.55
% of Variance	14.63	9.99	8.37	6.85	
Cumul. %	14.63	24.62	32.99	39.84	
Expl. Var	3.45	2.96	2.26	2.1	

Note: The values "0.00" represent no factor loading.

Table S21. Factor loadings of specific motor skills variables for the age group 15-17.

Variable	Factor 1	Factor 2	Factor 3	Factor 4	Communalities (h2)
**Horizontal Accuracy 15m**	0.38	0.00	0.00	0.00	0.34
**Horizontal Accuracy 20m**	0.36	0.00	0.00	0.00	0.43
**Horizontal Accuracy 25m**	0.38	0.00	0.00	0.00	0.30
**Vertical Accuracy - Hoop**	0.50	0.00	0.00	0.00	0.31
**Vertical Accuracy - Goal**	0.38	0.00	0.00	0.00	0.30
**Single Leg Ball Juggle**	0.62	0.00	0.00	0.00	0.59
**Alternately Leg Ball Juggle**	0.75	0.00	0.00	0.00	0.64
**Sprint with Ball 20m**	0.00	0.39	0.00	0.00	0.62
**Sprint with Ball 20m Flying Start**	0.00	0.43	0.00	0.00	0.67
**Sprint 20m**	0.00	0.49	0.00	0.00	0.55
**Slalom with Ball**	0.00	0.55	0.00	0.00	0.44
**Slalom without Ball**	0.00	0.24	0.00	0.00	0.39
**Semi-Circle Run with Ball**	0.00	0.64	0.00	0.00	0.37
**Semi-Circle Run without Ball**	0.00	0.51	0.00	0.00	0.14
**Dominant Leg Wall Pass**	0.63	0.00	0.00	0.00	0.28
**Non-Dominant Leg Wall Pass**	0.75	0.00	0.00	0.00	0.28
**Alternately Leg Wall Pass**	0.66	0.00	0.00	0.00	0.49
**Dominant Leg Parabolic Wall Pass**	0.65	0.00	0.00	0.00	0.51
**Non-Dominant Leg Parabolic Wall Pass**	0.43	0.00	0.00	0.00	0.51
**Alternately Leg Parabolic Wall Pass**	0.44	0.00	0.00	0.00	0.48
**Dominant Leg Explosive Power**	0.00	0.00	-0.63	0.00	0.50
**Non-Dominant Leg Explosive Power**	0.00	0.00	-0.76	0.00	0.57
**Head Explosive Power**	0.00	0.00	-0.74	0.00	0.46
% of Variance	15.67	11.00	8.84	6.56	
Cumul. %	15.67	26.66	35.50	42.06	
Expl. Var	4.2	2.85	2.39	1.91	

Note: The values "0.00" represent no factor loading.
